# Infant Infection With Respiratory Syncytial Virus Genotypes and Subsequent Childhood Asthma Risk

**DOI:** 10.1093/infdis/jiag104

**Published:** 2026-03-03

**Authors:** Christian Rosas-Salazar, Tebeb Gebretsadik, James D Chappell, R Stokes Peebles, William D Dupont, Meghan H Shilts, Samadhan J Jadhao, Larry J Anderson, Suman R Das, Tina V Hartert

**Affiliations:** Department of Pediatrics, Vanderbilt University Medical Center, Nashville, Tennessee, USA; Department of Biostatistics, Vanderbilt University Medical Center, Nashville, Tennessee, USA; Department of Pediatrics, Vanderbilt University Medical Center, Nashville, Tennessee, USA; Department of Medicine, Vanderbilt University Medical Center, Nashville, Tennessee, USA; Department of Biostatistics, Vanderbilt University Medical Center, Nashville, Tennessee, USA; Department of Medicine, Vanderbilt University Medical Center, Nashville, Tennessee, USA; Department of Pediatrics, Emory University School of Medicine and Children's Healthcare of Atlanta, Atlanta, Georgia, USA; Department of Pediatrics, Emory University School of Medicine and Children's Healthcare of Atlanta, Atlanta, Georgia, USA; Department of Medicine, Vanderbilt University Medical Center, Nashville, Tennessee, USA; Department of Pediatrics, Vanderbilt University Medical Center, Nashville, Tennessee, USA; Department of Medicine, Vanderbilt University Medical Center, Nashville, Tennessee, USA

**Keywords:** asthma, children, G gene, genotype, respiratory syncytial virus

## Abstract

Our objective was to test the hypothesis that respiratory syncytial virus (RSV) infection during infancy with genotypes containing previously reported G gene sequence duplications (G_dups_) is associated with an increased risk of childhood asthma. In a population-based, prospective, birth cohort of healthy, term children, we found that children with RSV-A G_dup+_ (adjusted odds ratio [aOR] = 2.00, 95%CI = 1.15–3.47) and RSV-B G_dup_  _+_ (aOR = 1.78, 95%CI = 1.01–3.11) infections during infancy had higher odds of 5-year current asthma than those without RSV infection during infancy. These findings are proof-of-concept evidence that RSV genetic variation is associated with variable childhood asthma risk.

## Teaser

Respiratory syncytial virus infection during infancy with genotypes containing previously reported G gene sequence duplications is associated with an increased risk of childhood asthma.

The body of research suggests that RSV infection during infancy is associated with an increased risk of childhood asthma [1]. Yet, a minority of infants infected with RSV will ultimately develop childhood asthma, suggesting that there are viral, host, and/or environmental factors that contribute to variable susceptibility to long-term health outcomes following RSV infection in the first year of life.

Because RSV G gene mutations have been postulated to impact the pathogenesis of this respiratory virus [2], we hypothesized that RSV infection during infancy with genotypes containing previously reported G gene sequence duplications (G_dups_) is associated with an increased risk of childhood asthma. To test this hypothesis, we leveraged a population-based, prospective, birth cohort of healthy, term children with close epidemiologic surveillance during each child's first RSV season, RSV whole-genome sequencing data from nasal washes collected during RSV infection during infancy, and serial follow-up for childhood asthma development.

## METHODS

Full details are available in the Supplementary Methods.

### Overview of the Study Design

The Infant Susceptibility to Pulmonary Infections and Asthma Following RSV Exposure Study (INSPIRE) recruited 1946 children near birth [1, 3]. Follow-up for the ascertainment of childhood asthma has been conducted annually. The Institutional Review Board of Vanderbilt University approved this study and one parent provided informed consent for their and their child's participation.

### Determination of RSV Infection During Infancy and RSV Whole-Genome Sequencing

Passive and active surveillance were performed during each child's first RSV season [1, 3]. If a child met pre-specified criteria for an acute respiratory infection, we collected a nasal wash that was used to detect RSV by reverse transcription-quantitative PCR (RT-qPCR). If the nasal wash was positive for RSV by RT-qPCR, we performed RSV whole-genome sequencing, genome assembly, and genotype assignment [4, 5]. In addition, we measured RSV serum antibody titers at age 1 year [1]. Children were classified as infected versus not infected with RSV during infancy based on the results of the RSV RT-qPCR and 1-year RSV serology as described elsewhere [1].

### Eligibility Criteria for the Current Study and Study Population

For the current study and based on our hypothesis, we included children enrolled in INSPIRE (1) with RSV infection during infancy and available RSV whole genome-sequencing data, and (2) without RSV infection during infancy to serve as an RSV-negative comparator group (Supplementary Figure 1).

### Genotype of the RSV Infection During Infancy

The genotype of the RSV infection during infancy was categorized using the available RSV whole genome-sequencing data into mutually exclusive groups based on the antigenic subgroup (RSV-A vs RSV-B) and the presence of previously reported G_dups_ (yes vs no [hereinafter termed G_dup+_ and G_dup–_, respectively]) [2, 4].

### Definition of Outcomes

Our primary, secondary, and exploratory outcomes were 5-year current asthma, the respiratory severity score (RSS), and the 5-year childhood asthma phenotype (allergic or nonallergic), respectively, as defined in the Supplementary Methods [1].

### Statistical Analyses

To examine the associations of interest, we used unadjusted and adjusted linear regression, binary logistic regression, or multinomial logistic regression. The adjusted models included the child's sex, race and ethnicity, maternal asthma, ever breastfeeding, daycare attendance during infancy, and (for the outcome of the RSS only) age at RSV infection as base covariates, which were selected a priori based on published literature and by creating a causal directed acyclic graph (Supplementary Figure 2). For our primary outcome, we also (1) built supplementary models by either replacing base covariates with proxy covariates or by including additional covariates, and (2) tested for interactions between genotype of the RSV infection and the child's sex, race and ethnicity, and maternal asthma in separate models while adjusting for base covariates. A two-sided *P*-value <.05 was considered statistically significant. Statistical analyses were performed using R.

## RESULTS

One thousand one hundred and two (56.63%) of the 1946 eligible children met eligibility criteria for the current study, including 305 (27.68%) children with RSV infection during infancy (Supplementary Figure 1). The circulation of the different RSV genotypes varied by RSV season (Supplementary Figure 3). There were no RSV infections during infancy with the RSV-B G_dup–_ genotype.

Children with RSV-A G_dup–_ infection during infancy were more likely to be older at enrollment, male, enrolled in 2012, and to have attended daycare during infancy than children in other groups (Table 1). Children with RSV-A G_dup+_ infection during infancy were more likely to have lived with another child aged <6 years at home during infancy than children in other groups (Table 1). The baseline characteristics of children who were included and not included in the current study are shown in Supplementary Table 1.

**Table 1.** Baseline characteristics of children included in the current study by genotype of the RSV infection during infancy.^a,b^

	No RSV Infection During Infancy (*n* = 797 [72.32%])	RSV Infection During Infancy With The RSV-A G_dup+_ Genotype (*n* = 124 [11.25%)	RSV Infection During Infancy With The RSV-A G_dup−_ Genotype (*n* = 60 [5.44%])	RSV Infection During Infancy With The RSV-B G_dup+_ Genotype (*n* = 121 [10.98%])	All (*n* = 1102)
Age at enrollment (days)	51 (15, 75)	60 (16, 82)	63 (28, 111)	37 (15, 70)^c^	53 (15, 76)
Age at RSV infection (months)	—	4 (2, 5)	5 (3, 6)	4 (2, 5)	—
Female sex	391 (49%)	60 (48%)	17 (28%)	59 (49%)^c^	527 (48%)
Race and ethnicity					
Black non-Hispanic	116 (15%)	20 (16%)	6 (10%)	19 (16%)	161 (15%)
White non-Hispanic	551 (69%)	84 (68%)	42 (70%)	86 (71%)	763 (69%)
Hispanic	65 (8%)	12 (10%)	7 (12%)	7 (6%)	91 (8%)
Other	65 (8%)	8 (6%)	5 (8%)	9 (7%)	87 (8%)
Enrollment year (first RSV season)					
2012 (2012–2013)	340 (43%)	81 (65%)	58 (97%)	22 (18%)^c^	501 (45%)
2013 (2013–2014)	457 (57%)	43 (35%)	2 (3%)	99 (82%)	601 (55%)
Gestational age (weeks)	39 (39, 40)	39 (39, 40)	39 (39, 40)	39 (38, 40)	39 (39, 40)
Birth weight (grams)	3405 (3,120, 3717)	3433 (3,121, 3717)	3575 (3,299, 3866)	3405 (3,125, 3710)	3433 (3,121, 3746)
Birth by cesarean section	233 (29%)	37 (30%)	27 (45%)	33 (27%)	330 (30%)
Ever breastfeeding	653 (82%)	91 (75%)	51 (88%)	93 (80%)	888 (81%)
Daycare attendance during infancy	234 (29%)	45 (38%)	28 (52%)	54 (47%)^c^	361 (33%)
Presence of another child aged <6 years at home during infancy	353 (44%)	79 (64%)	34 (57%)	66 (55%)^c^	532 (48%)
Maternal asthma	160 (20%)	21 (17%)	13 (22%)	23 (19%)	217 (20%)
Exposure to secondhand smoke in utero or during early infancy	162 (20%)	33 (27%)	8 (13%)	29 (24%)	232 (21%)
Type of insurance					
Federal or state	402 (50%)	61 (49%)	24 (40%)	65 (54%)	552 (50%)
Private	384 (48%)	61 (49%)	34 (57%)	56 (46%)	535 (49%)
Other or unknown	11 (1%)	2 (2%)	2 (3%)	0 (0%)	15 (1%)
Socioeconomic status theme of the social vulnerability index	0.54 (0.36, 0.74)	0.55 (0.31, 0.72)	0.55 (0.35, 0.71)	0.49 (0.32, 0.68)	0.53 (0.34, 0.72)

Abbreviations: G_dup_, G gene sequence duplication; RSV, Respiratory syncytial virus.

^a^Data presented as median (interquartile range) for continuous variables or frequencies (%) for categorical variables.

^b^Statistical analyses included children with complete data.

^c^ *P* < .05 for the comparison between the groups using Kruskal-Wallis or Pearson chi-squared tests as appropriate.

**Figure 1.**
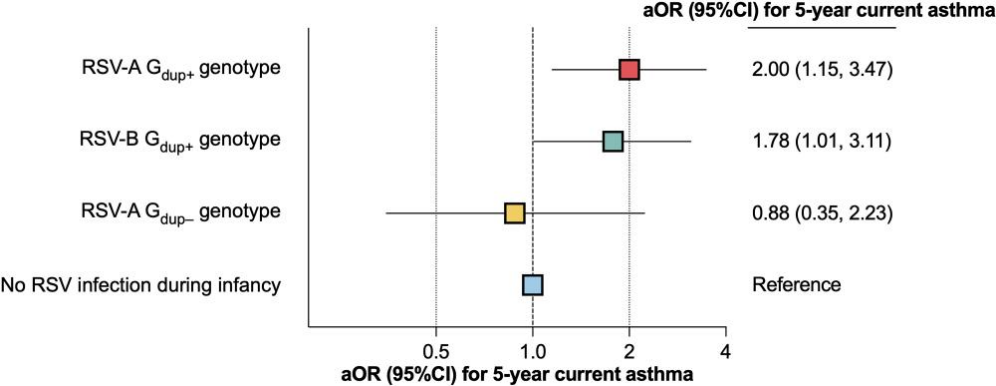
The association of genotype of the RSV infection during infancy with 5-year current asthma. The plot estimates were obtained from a binary logistic regression model that included the child's sex, race and ethnicity, maternal asthma, ever breastfeeding, and daycare attendance during infancy as covariates. The aOR (squares) and corresponding 95%CI (adjacent lines) for each genotype of the RSV infection during infancy are shown. Children without RSV infection during infancy were included as the reference group for the model. Abbreviations: aOR, Adjusted odds ratio; CI, Confidence interval; G_dup_, G gene sequence duplication; RSV, Respiratory syncytial virus.

The 5-year follow-up visit rate for children included in the current study was 797/1102 (72.32%). The proportion of 5-year current asthma was 22/85 (25.88%) in children with RSV-A G_dup+_ infection during infancy, 21/87 (24.14%) in children with RSV-B G_dup+_ infection during infancy, 6/38 (15.79%) in children with RSV-A G_dup−_ infection during infancy, and 91/587 (15.50%) in children without RSV infection during infancy (*P* = .04). In adjusted models including base covariates, there was a significant association of genotype of the RSV infection during infancy with the odds of 5-year current asthma (overall *P* = .03). Children with RSV-A G_dup+_ and RSV-B G_dup+_ infections during infancy had higher odds of 5-year current asthma than those without RSV infection during infancy, whereas those with RSV-A G_dup−_ infection during infancy did not (Figure 1 and Supplementary Table 2). In supplementary models, we obtained overall similar results when replacing base covariates with proxy covariates or when including additional covariates. However, only the estimates of the association among children with RSV-A G_dup+_ infection during infancy remained consistent across all supplementary models (Supplementary Table 2). The association of genotype of the RSV infection during infancy and 5-year current asthma was not modified by the child's sex, race and ethnicity, or maternal asthma (*P* > .05 for all interaction terms).

The median (interquartile range) RSS was 3 (2–4) in children with RSV-A G_dup+_ infection during infancy, 3 (2–4) in children with RSV-B G_dup+_ infection during infancy, and 2 (2–4) in children with RSV-A G_dup−_ infection during infancy (*P* = .4). There was no association of genotype of the RSV infection during infancy with the RSS in adjusted models including base covariates (Supplementary Table 3).

The proportion of 5-year current asthma phenotypes differed by genotype of the RSV infection during infancy (*P* = .006). In adjusted models including base covariates, children with RSV-A G_dup+_ infection during infancy had higher odds of 5-year current nonallergic asthma than those without RSV infection during infancy, whereas the odds of 5-year current nonallergic asthma were not increased among children with RSV-B G_dup+_ or RSV-A G_dup−_ infection during infancy. In contrast, the odds of 5-year current allergic asthma were not increased among children with RSV-A G_dup+_, RSV-B G_dup+_, or RSV-A G_dup−_ (Supplementary Table 4).

## DISCUSSION

In our study, we found that children with RSV-A G_dup+_ infection during infancy had approximately 2-fold increased odds of childhood asthma than those with RSV infection during infancy with genotypes lacking previously reported G_dups_ or those without RSV infection during infancy. Our findings are important as they (1) are proof-of-concept evidence that RSV genetic variation is associated with variable childhood asthma risk, and (2) may partly explain the difference in asthma susceptibility among children with RSV infection during infancy. To our knowledge, this the first study to show that individual RSV genotypes can differentially impact the risk of childhood asthma.

In line with our prior studies, we found that multiple RSV genotypes were co-circulating during the 2012–2014 RSV seasons in middle Tennessee [4, 6]. Our results of an absence of circulating RSV-B G_dup−_ genotypes is expected, as the G_dup_ became fixed across RSV-B lineages after 1999 [4]. The G_dup_ was not reported among RSV-A lineages until 2010, but the insertion is present as an exact, tandem, in-frame duplication of the same region in both RSV-A and RSV-B, possibly because the G_dup_ provides some type of selective advantage to the two antigenic subgroups and helps evade herd immunity [2, 4].

Because of our study's observational design, we can only hypothesize the mechanisms through which RSV infection during infancy with genotypes containing previously reported G_dups_ could confer a higher risk of childhood asthma. The G glycoprotein is the principal viral attachment protein and genetic variations in the G gene have been implicated in RSV pathogenesis in numerous pre-clinical and some human studies. For example, experiments in murine models have shown that administration of anti-G glycoprotein antibodies prior to and/or after RSV infection leads to decreased viral titers, airway inflammation, weight loss, airway mucous production, work of breathing, and/or airway hyper responsiveness [2, 7–9]. The results of in vitro studies also suggest that the G glycoprotein has wide ranging effects on the host's innate and adaptive immune responses [2]. In regard to the specific functional effects of G_dups_, one study that generated recombinant viruses demonstrated that RSV-B G_dup_  _+_ genotypes can more efficiently attach to the host's cell surface and better replicate than RSV-B G_dup−_ genotypes [10]. Similarly, a study that generated lentiviral pseudo particles harboring G glycoproteins from RSV-A G_dup+_ and RSV-A G_dup−_ genotypes indicated that the G_dup_ has a higher infectivity and may offer a selective advantage [11]. Likewise, we previously identified unique G gene variants associated with RSV persistence and an increased acute disease severity among children infected with RSV during infancy [5, 12]. On the other hand, studies examining whether G_dups_ impact the acute disease severity of the RSV infection during infancy have yielded conflicting results [13, 14].

Our findings suggest that children with RSV-A G_dup+_ infection during infancy are at a higher risk of developing a nonallergic childhood asthma phenotype. Because of the small sample sizes for some subgroups, these statistical analyses should be considered exploratory. Nonetheless, these results are consistent with our previous studies showing an increased proportion of nonallergic childhood asthma phenotypes among children with RSV infection during infancy [1].

Our study has multiple strengths, including (1) the novel a priori hypothesis, (2) the inclusion of children without RSV infection during infancy as the reference group, whereas most studies examining the association of RSV infection during infancy with childhood asthma have not had a true control group, and (3) the collection of samples during a period when both RSV-A G_dup+_ and RSV-A G_dup−_ genotypes were co-circulating in our region and before the RSV-A G_dup+_ genotype became the predominant circulating strain worldwide [4, 6]. We should also acknowledge certain limitations. First, we cannot exclude misclassification of children categorized as not infected with RSV during infancy. However, the serologic method we used to determine the RSV-negative comparator group performs well compared with other similar laboratory assays [15]. Second, it is difficult to ascertain childhood asthma in younger children as they frequently cannot perform objective testing. It is also possible that individual RSV genotypes could differentially impact childhood asthma phenotypes not considered in the current study (eg, early-transient vs persistent wheeze), which will need to be assessed in future studies. Third, we used antigenic subgroups and the presence of previously reported G_dups_ to categorize RSV genotypes based on our hypothesis and because these represent the major new viral strains emerging over the last several decades [2, 4]. However, it is conceivable that the distinct risks of childhood asthma among RSV genotypes could be attributed to other genetic variations not considered in the current study. Last, unmeasured confounding could not be completely ruled out from influencing our results.

In summary, our findings reveal that RSV infection during infancy with genotypes containing previously reported G_dups_ is associated with an increased risk of childhood asthma. While RSV genotypes lacking previously reported G_dups_ are no longer circulating, our findings emphasize the need for continuing surveillance of RSV evolutionary dynamics. This is particularly important in the context of the recently introduced RSV prevention products, which are highly effective in reducing acute disease severity, but do not impact the risk of RSV infection during infancy and could lead to the emergence of more pathogenic RSV genotypes. Furthermore, our results underscore the importance of considering individual RSV genotypes when examining the association of RSV infection during infancy with long-term pulmonary disease morbidity.

## Supplementary Material

jiag104_Supplementary_Data

## Supplementary Data

Supplementary materials are available at *The Journal of Infectious Diseases* online (http://jid.oxfordjournals.org/). Supplementary materials consist of data provided by the author that are published to benefit the reader. The posted materials are not copyedited. The contents of all supplementary data are the sole responsibility of the authors. Questions or messages regarding errors should be addressed to the author.

## References

[1] Rosas-Salazar C, Chirkova T, Gebretsadik T, et al Respiratory syncytial virus infection during infancy and asthma during childhood in the USA (INSPIRE): a population-based, prospective birth cohort study. Lancet 2023; 401:1669–80. 37086744 10.1016/S0140-6736(23)00811-5 PMC10367596

[2] Anderson LJ, Jadhao SJ, Paden CR, Tong S. Functional features of the respiratory syncytial virus G protein. Viruses 2021; 14:13. 34372490 10.3390/v13071214 PMC8310105

[3] Larkin EK, Gebretsadik T, Moore ML, et al Objectives, design and enrollment results from the infant susceptibility to pulmonary infections and asthma following RSV exposure study (INSPIRE). BMC Pulm Med 2015; 15:45. 26021723 10.1186/s12890-015-0040-0 PMC4506623

[4] Schobel SA, Stucker KM, Moore ML, et al Respiratory syncytial virus whole-genome sequencing identifies convergent evolution of sequence duplication in the C-terminus of the G gene. Sci Rep 2016; 6:26311. 27212633 10.1038/srep26311 PMC4876326

[5] Lawless D, McKennan CG, Das SR, et al Viral genetic determinants of prolonged respiratory syncytial virus infection among infants in a healthy term birth cohort. J Infect Dis 2022; 227:1194–1202. 10.1093/infdis/jiac442 PMC10175068 36375000

[6] Tan Y, Shilts MH, Rosas-Salazar C, et al Influence of sex on respiratory syncytial virus genotype infection frequency and nasopharyngeal microbiome. J Virol 2023; 97:e0147222. 36815771 10.1128/jvi.01472-22 PMC10062153

[7] Boyoglu-Barnum S, Gaston KA, Todd SO, et al A respiratory syncytial virus (RSV) anti-G protein F(ab’)2 monoclonal antibody suppresses mucous production and breathing effort in RSV rA2-line19F-infected BALB/c mice. J Virol 2013; 87:10955–67. 23885067 10.1128/JVI.01164-13 PMC3807296

[8] Caidi H, Miao C, Thornburg NJ, Tripp RA, Anderson LJ, Haynes LM. Anti-respiratory syncytial virus (RSV) G monoclonal antibodies reduce lung inflammation and viral lung titers when delivered therapeutically in a BALB/c mouse model. Antiviral Res 2018; 154:149–57. 29678551 10.1016/j.antiviral.2018.04.014 PMC8063470

[9] Han J, Takeda K, Wang M, et al Effects of anti-g and anti-f antibodies on airway function after respiratory syncytial virus infection. Am J Respir Cell Mol Biol 2014; 51:143–54. 24521403 10.1165/rcmb.2013-0360OC PMC4091856

[10] Hotard AL, Laikhter E, Brooks K, Hartert TV, Moore ML. Functional analysis of the 60-nucleotide duplication in the respiratory syncytial virus Buenos Aires strain attachment glycoprotein. J Virol 2015; 89:8258–66. 26018171 10.1128/JVI.01045-15 PMC4524256

[11] Cui G, Liu H, Li X, Ming L. Preliminary functional and phylogeographic analyses of the 72 nucleotide duplication region in the emerging human respiratory syncytial virus ON1 strain attachment glycoprotein gene. Biomed Pharmacother 2020; 123:109800. 31901716 10.1016/j.biopha.2019.109800

[12] Human S, Hotard AL, Rostad CA, et al A respiratory syncytial virus attachment gene variant associated with more severe disease in infants decreases fusion protein expression, which may facilitate immune evasion. J Virol 2020; 95:e01201-20. 33115881 10.1128/JVI.01201-20 PMC7944440

[13] Midulla F, Nenna R, Scagnolari C, et al How respiratory syncytial virus genotypes influence the clinical course in infants hospitalized for bronchiolitis. J Infect Dis 2019; 219:526–34. 30204889 10.1093/infdis/jiy496

[14] Vandini S, Biagi C, Lanari M. Respiratory syncytial virus: the influence of serotype and genotype variability on clinical course of infection. Int J Mol Sci 2017; 18:1717. 28783078 10.3390/ijms18081717 PMC5578107

[15] Jadhao SJ, Ha B, McCracken C, et al Performance evaluation of antibody tests for detecting infant respiratory syncytial virus infection. J Med Virol 2021; 93:3439–45. 33325064 10.1002/jmv.26736 PMC8046717

