# Supplementary material for: Infant Infection With Respiratory Syncytial Virus Genotypes and Subsequent Childhood Asthma Risk
Source: J Infect Dis. 2026 Mar 3;234(1):e34–9. doi: 10.1093/infdis/jiag104 (PMC13431657; doi:10.1093/infdis/jiag104)
Supplement: jiag104_Supplementary_Data [file jiag104_supplementary_data.zip › crs_rsv_gdup_supplementary_methods_R1.docx]

**Infant Infection with Respiratory Syncytial Virus Genotypes and Subsequent Childhood Asthma Risk**

**Supplementary Methods**

Christian Rosas-Salazar, MD, MPH, Tebeb Gebretsadik, MPH, James D. Chappell, MD, PhD, R. Stokes Peebles Jr., MD, William D. Dupont, PhD, Meghan H. Shilts, MS, Samadhan J. Jadhao, PhD, Larry J. Anderson, MD, Suman R. Das, PhD, and Tina V. Hartert, MD, MPH

Table of Contents

Supplementary Methods ……………………………………………………………. Page 2

Supplementary References …………………………………………………………. Page 8

**Supplementary Methods:**

Overview of the INSPIRE Study Design

The full methods for the Infant Susceptibility to Pulmonary Infections and Asthma Following Respiratory Syncytial Virus Exposure Study (INSPIRE) have been previously reported [1, 2]. INSPIRE recruited 1,946 healthy, term children near birth from 11 participating pediatric practices across middle Tennessee. Children had to be born between June and December of 2012 or June and December of 2013 and were thus ≤6 months of age at the beginning of their first respiratory syncytial virus (RSV) season (November to March in our region) [3]. The catchment zone encompassed urban, suburban, and rural areas. Follow-up for the ascertainment of childhood asthma has been conducted annually. The Institutional Review Board of Vanderbilt University approved this study and one parent provided informed consent for their and their child’s participation (IRB # 111299).

Determination of RSV Infection During Infancy and RSV Whole-Genome Sequencing

In all children enrolled in INSPIRE, we conducted passive and active surveillance during each child’s first RSV season by 1) performing bi-weekly phone, email, and/or in person follow-up using structured parental surveys, 2) frequently educating and reminding parents to call us at the onset of any symptom suggestive of an acute respiratory infection (ARI), and 3) approaching children seen at participating pediatric practices for an unscheduled visit. An ARI was defined as parental report of 1) one of the following major symptoms or diagnoses: wheezing, difficulty in breathing, or presence of a positive RSV test, or 2) any two of the following minor symptoms or diagnoses: fever, runny nose/nasal congestion/snotty nose, cough, ear infection, or hoarse cry. If a child met these pre-specified criteria for an ARI, we conducted an in-person respiratory illness visit at which time we administered a parental questionnaire, performed a physical exam, collected a nasal wash, and —in those who required a health care encounter— completed a structured medical chart review. The nasal wash was used to detect RSV by reverse transcription-quantitative PCR (RT-qPCR) as per a published protocol [4]. If the nasal wash was positive for RSV by RT-qPCR, we performed RSV whole-genome sequencing, genome assembly, and genotype assignment as previously described [5, 6]. In addition, we collected blood samples from all participating children at age one year and measured RSV serum antibody titers by an enzyme-linked immunosorbent assay [7]. This technique uses RSV-A and RSV-B lysate antigens produced in a human epithelial type 2 (HEp-2) cell line and detects serum antibodies against a range of RSV proteins (including the F, G, N, and P proteins). Serum antibody titers ≤200 to either RSV-A or RSV-B lysates were considered negative. Children were classified as infected *vs.* not infected with RSV during infancy based on the results of the RSV RT-qPCR and one-year RSV serology as described elsewhere [2].

Eligibility Criteria for the Current Study and Study Population

For the current study and based on our hypothesis, we included children enrolled in INSPIRE 1) with RSV infection during infancy (as determined by a nasal wash positive for RSV by RT-qPCR) and available RSV whole genome-sequencing data, and 2) without RSV infection during infancy (as determined by no nasal wash positive for RSV by RT-qPCR and a negative one-year RSV serology) to serve as an RSV-negative comparator group. Children enrolled in INSPIRE who had 1) a nasal wash positive for RSV by RT-qPCR but no available RSV whole-genome data, 2) no nasal wash positive for RSV by RT-qPCR and no one-year blood sample available, or 3) no nasal wash positive for RSV by RT-qPCR but a positive one-year RSV serology (and who likely had an asymptomatic or minimally symptomatic RSV infection) [2] were excluded from the current study as we could not categorize the genotype of the RSV infection during infancy (**Supplementary Figure 1**).

Genotype of the RSV Infection during Infancy

The genotype of the RSV infection during infancy was categorized using the available RSV whole genome-sequencing data into mutually exclusive groups based on the antigenic subgroup (RSV-A *vs.* RSV-B) and the presence of previously reported G_dups_ (yes *vs.* no [hereinafter termed G_dup+_ and G_dup–_, respectively]) [5, 8-10]. If a child had more than one nasal wash positive for RSV by RT-qPCR (n =19/361 [5.26%]), only the first one was used to categorize the genotype of the RSV infection during infancy.

Definition of Outcomes

Our primary outcome was 5-year current asthma, which was defined as parental report of 1) physician-diagnosed asthma or use of asthma medications (including short-acting beta agonists, inhaled steroids, leukotriene receptor antagonists, or long-acting beta agonists) at any time point prior to age 5 years, and 2) any of the following occurring in the 12 months prior to the 5-year follow-up visit: asthma symptoms (such as “any wheezing or whistling in the chest”, “chest sounded wheezy during or after exercise”, and “dry cough at night apart from a cough associated with a cold or chest infection”), asthma-related systemic steroid use, or acute health care utilization for asthma (including urgent care encounters emergency department visits, or hospitalizations) [2].

Our secondary outcome was the acute disease severity of the RSV infection, which was ascertained using the respiratory severity score (RSS). The RSS is an ordinal scale based on respiratory rate, flaring or retractions, heart rate, and wheezing that was slightly modified from other composite scores derived for ARIs [11, 12]. It ranges from 0 to 12, with higher values indicating more severe disease.

Our exploratory outcome was the 5-year current asthma phenotype (allergic or non-allergic), which was ascertained using 1) the aforementioned definition of 5-year current asthma, and 2) evidence of sensitization to common aeroallergens at age 3 years [2]. Evidence of aeroallergen sensitization at age 3 years was determined by either 2a) a positive skin prick testing (a wheal ≥3 mm larger than negative control) to common aeroallergens (weeds, grasses, trees, dogs, cats, dust mites, and molds) during the 3-year follow-up visit, or 2b) a positive (≥0.35 kU/L) ImmunoCAP Phadiatop specific IgE panel (ThermoFisher Scientific, Massachusetts, United States) in blood obtained during the 3-year follow-up visit (only performed if skin prick testing could not be done due to parent or child refusal or could not be interpreted due to inadequate controls).

The primary, secondary, and exploratory outcomes were assessed in all children regardless of genotype of the RSV infection during infancy unless they were lost to follow-up.

Statistical Analyses

Descriptive statistics are presented as median (interquartile range) for continuous variables and frequencies (%) for categorical variables. To compare baseline characteristics of participating children by genotype of the RSV infection during infancy, we used Kruskal-Wallis or Pearson chi-squared tests. To examine the association of genotype of the RSV infection during infancy with 5-year current asthma and the 5-year current asthma phenotype, we used unadjusted and adjusted binary and multinomial logistic regression models and included children without RSV infection during infancy as the reference category. To examine the association of genotype of the RSV infection during infancy with the RSS, we used unadjusted and adjusted linear regression models and included children with RSV infection during infancy with the RSV-A G_dup–_ genotype as the reference category. We *a priori* selected base covariates to be included in the adjusted models based on published literature and by creating a causal directed acyclic graph (**Supplementary Figure 2**). These included the child’s sex, race and ethnicity, maternal asthma, ever breastfeeding, daycare attendance during infancy, and (for the outcome of the RSS only) age at RSV infection [2, 13]. For our primary outcome, we also 1) built supplementary models by either replacing the base covariates with proxy covariates or by including additional covariates (such as the presence of another child younger than 6 years, enrollment year, the socioeconomic status theme of the social vulnerability index, and exposure to secondhand smoke *in utero* or during early infancy [14]), and 2) tested for interactions between genotype of the RSV infection and the child’s sex, race and ethnicity, or maternal asthma in separate models while adjusting for base covariates. Because we had a low proportion of missing covariates, all statistical analyses were conducted in children with complete data. A two-sided p-value <0.05 was considered statistically significant. Statistical analyses were performed using R version 4.4.1 [15]. Figures were created using the R *ggplot2* package, GraphPad Prism version 8.4.3 (available at: <https://www.graphpad.com/>), Biorender (available at: <https://biorender.com/>), drawio (available at: <https://app.diagrams.net/>), and DAGitty (available at: <http://www.dagitty.net/>). Minor aesthetic edits to figures (such as paneling, text insertion, or label formatting) were made with Inkscape version 1.0.2 (available at: <https://inkscape.org/>).

**Supplementary References:**

1. Larkin EK, Gebretsadik T, Moore ML, et al. Objectives, design and enrollment results from the Infant Susceptibility to Pulmonary Infections and Asthma Following RSV Exposure Study (INSPIRE). BMC Pulm Med **2015**; 15:45.

2. Rosas-Salazar C, Chirkova T, Gebretsadik T, et al. Respiratory syncytial virus infection during infancy and asthma during childhood in the USA (INSPIRE): a population-based, prospective birth cohort study. Lancet **2023**; 401:1669–80.

3. Haynes AK, Prill MM, Iwane MK, Gerber SI. Respiratory syncytial virus—United States, July 2012–June 2014. Morbidity and Mortality Weekly Report **2014**; 63:1133.

4. Kodani M, Yang G, Conklin LM, et al. Application of TaqMan low-density arrays for simultaneous detection of multiple respiratory pathogens. J Clin Microbiol **2011**; 49:2175–82.

5. Schobel SA, Stucker KM, Moore ML, et al. Respiratory Syncytial Virus whole-genome sequencing identifies convergent evolution of sequence duplication in the C-terminus of the G gene. Scientific reports **2016**; 6:26311.

6. Lawless D, McKennan CG, Das SR, et al. Viral genetic determinants of prolonged respiratory syncytial virus infection among infants in a healthy term birth cohort. J Infect Dis **2022**.

7. Jadhao SJ, Ha B, McCracken C, et al. Performance evaluation of antibody tests for detecting infant respiratory syncytial virus infection. J Med Virol **2021**; 93:3439–45.

8. Eshaghi A, Duvvuri VR, Lai R, et al. Genetic variability of human respiratory syncytial virus A strains circulating in Ontario: a novel genotype with a 72 nucleotide G gene duplication. PloS one **2012**; 7:e32807.

9. Trento A, Galiano M, Videla C, et al. Major changes in the G protein of human respiratory syncytial virus isolates introduced by a duplication of 60 nucleotides. J Gen Virol **2003**; 84:3115–20.

10. Tan Y, Shilts MH, Rosas-Salazar C, et al. Influence of Sex on Respiratory Syncytial Virus Genotype Infection Frequency and Nasopharyngeal Microbiome. J Virol **2023**; 97:e0147222.

11. McCallum GB, Morris PS, Wilson CC, et al. Severity scoring systems: are they internally valid, reliable and predictive of oxygen use in children with acute bronchiolitis? Pediatr Pulmonol **2013**; 48:797–803.

12. Rodriguez H, Hartert TV, Gebretsadik T, Carroll KN, Larkin EK. A simple respiratory severity score that may be used in evaluation of acute respiratory infection. BMC research notes **2016**; 9:85.

13. Abreo A, Gebretsadik T, Stone CA, Hartert TV. The impact of modifiable risk factor reduction on childhood asthma development. Clin Transl Med **2018**; 7:15.

14. Flanagan BE, Gregory EW, Hallisey EJ, Heitgerd JL, Lewis B. A social vulnerability index for disaster management. J Homel Secur Emerg **2011**; 8:0000102202154773551792.

15. R Development Core Team. R: A language and environment for statistical computing. Vienna, Austria: R Foundation for Statistical Computing, **2006**.
