# Supplementary material for: Infant Infection With Respiratory Syncytial Virus Genotypes and Subsequent Childhood Asthma Risk
Source: J Infect Dis. 2026 Mar 3;234(1):e34–9. doi: 10.1093/infdis/jiag104 (PMC13431657; doi:10.1093/infdis/jiag104)
Supplement: jiag104_Supplementary_Data [file jiag104_supplementary_data.zip › crs_rsv_gdup_supplementary_table_1_R1.docx]

| **Supplementary Table 1.** Baseline characteristics of children enrolled in the Infant Susceptibility to Pulmonary Infections and Asthma Following RSV Exposure Study (INSPIRE) who were included and not included in the current study.^*†^ | | | |
| --- | --- | --- | --- |
| Baseline characteristics | Included in the current study | | All (n=1,946) |
|  | Yes (n=1,102 [56.63%]) | No (n=844 [43.37%]) |  |
| Age at enrollment (days) | 53 (15, 76) | 58 (17, 82)^‡^ | 55 (16, 78) |
| Female sex | 527 (48%) | 399 (47%) | 926 (48%) |
| Race and ethnicity  Black non-Hispanic  White non-Hispanic  Hispanic  Other | 161 (15%)  763 (69%)  91 (8%)  87 (8%) | 182 (22%)^‡^  504 (60%)  79 (9%)  79 (9%) | 343 (18%)  1,267 (65%)  170 (9%)  166 (9%) |
| Enrollment year (first RSV season)  2012 (2012-2013)  2013 (2013-2014) | 501 (45%)  601 (55%) | 357 (42%)  487 (58%) | 858 (44%)  1,088 (56%) |
| Gestational age (weeks) | 39 (39-40) | 39 (38-40) | 39 (39-40) |
| Birth weight (grams) | 3,433 (3,121, 3,746) | 3,405 (3,093, 3,740) | 3,405 (3,120, 3,740) |
| Birth by cesarean section | 330 (30%) | 281 (33%) | 611 (31%) |
| Ever breastfeeding | 888 (81%) | 640 (81%) | 1,528 (81%) |
| Daycare attendance during infancy | 361 (33%) | 229 (34%) | 590 (34%) |
| Presence of another child aged <6 years at home during infancy | 532 (48%) | 451 (53%)^‡^ | 983 (51%) |
| Maternal asthma | 217 (20%) | 162 (19%) | 379 (19%) |
| Exposure to secondhand smoke *in utero* or during early infancy | 232 (21%) | 193 (23%) | 425 (22%) |
| Type of insurance  Federal or state  Private  Other or unknown | 552 (50%)  535 (49%)  15 (1%) | 503 (60%)^‡^  332 (39%)  7 (1%) | 1,055 (54%)  867 (45%)  22 (1%) |
| Socioeconomic status domain of the social vulnerability index | 0.53 (0.34, 0.72) | 0.58 (0.38, 0.75)^‡^ | 0.56 (0.36, 0.75) |
| *Definition of abbreviations:* RSV = Respiratory syncytial virus.  ^*^Data presented as median (interquartile range) for continuous variables or number (%) for categorical variables.  ^†^Statistical analyses included children with complete data.  ^‡^p<0.05 for the comparison between the groups using a Mann-Whitney U or Pearson chi-squared test as appropriate. | | | |
