# Supplementary material for: Infant Infection With Respiratory Syncytial Virus Genotypes and Subsequent Childhood Asthma Risk
Source: J Infect Dis. 2026 Mar 3;234(1):e34–9. doi: 10.1093/infdis/jiag104 (PMC13431657; doi:10.1093/infdis/jiag104)
Supplement: jiag104_Supplementary_Data [file jiag104_supplementary_data.zip › crs_rsv_gdup_supplementary_table_2_R1.docx]

| **Supplementary Table 2.** The association of genotype of the RSV infection during infancy with 5-year current asthma.^*†^ | | | | | | | | |
| --- | --- | --- | --- | --- | --- | --- | --- | --- |
| Genotype of the RSV infection during infancy |  | Unadjusted model  (n=140/797 [17.57%]) |  | Adjusted models^‡^ | | | | |
|  |  |  |  | Base model  (n=140/794 [17.63%])^‡^ | Supplementary model 1 (n=140/795 [17.61%])^§^ | Supplementary model 2 (n=140/794 [17.63%])^ll^ | Supplementary model 3 (n=139/792 [17.55%])^**^ | Supplementary model 4 (n=_/_ [_%])^††^ |
| No RSV infection during infancy |  | Reference |  | Reference | Reference | Reference | Reference | Reference |
| RSV infection during infancy with the RSV-A G_dup–_ genotype |  | 1.02 (0.42, 2.51) |  | 0.88 (0.35, 2.23) | 0.91 (0.36, 2.30) | 0.84 (0.32, 2.19) | 0.84 (0.33, 2.14) | 0.90 (0.35, 2.28) |
| RSV infection during infancy with the RSV-B G_dup+_ genotype |  | 1.73 (1.01, 2.97) |  | 1.78 (1.01, 3.11) | 1.83 (1.05, 3.19) | 1.82 (1.03, 3.22) | 1.70 (0.96, 3.02) | 1.78 (1.01, 3.11) |
| RSV infection during infancy with the RSV-A G_dup+_ genotype |  | 1.90 (1.12, 3.25) |  | 2.00 (1.15, 3.47) | 2.05 (1.18, 3.57) | 1.96 (1.12, 3.43) | 2.04 (1.17, 3.55) | 1.95 (1.12, 3.41) |
| Overall p-value |  | 0.04 |  | 0.03 | 0.02 | 0.03 | 0.03 | 0.04 |
| *Definition of abbreviations:* CI = Confidence interval, G_dup_ = G gene sequence duplication, OR = Odds ratio, RSV = Respiratory syncytial virus.  ^*^Table estimates were obtained from unadjusted and adjusted binary logistic regression models and are shown as OR (95%CI). The overall p-values for the categorical exposure term and the number of children with outcome / total number of children included in each model (n [%]) are also shown. For all models, the reference group included children without RSV infection during infancy.  ^†^Statistical analyses were conducted in children with complete data.  ^‡^The adjusted base model included the child’s sex, race and ethnicity, maternal asthma, ever breastfeeding, and daycare attendance during infancy as covariates.  ^§^The adjusted supplementary model 1 included the same covariates as the adjusted base model but replaces daycare attendance during infancy with the presence of another child younger than 6 years as a covariate.  ^ll^The adjusted supplementary model 2 included the same covariates as the adjusted base model plus enrollment year as an additional covariate.  ^**^The adjusted supplementary model 3 included the same covariates as the adjusted base model plus the socioeconomic status theme of the social vulnerability index as an additional covariate.  ^††^The adjusted supplementary model 4 included the same covariates as the adjusted base model plus exposure to secondhand smoke *in utero* or during early infancy as an additional covariate. | | | | | | | | |
