# Supplementary material for: Infant Infection With Respiratory Syncytial Virus Genotypes and Subsequent Childhood Asthma Risk
Source: J Infect Dis. 2026 Mar 3;234(1):e34–9. doi: 10.1093/infdis/jiag104 (PMC13431657; doi:10.1093/infdis/jiag104)
Supplement: jiag104_Supplementary_Data [file jiag104_supplementary_data.zip › crs_rsv_gdup_supplementary_table_3_R1.docx]

| **Supplementary Table 3.** The association of genotype of the RSV infection during infancy with the respiratory severity score.^*†^ | | | | |
| --- | --- | --- | --- | --- |
| Genotype of the RSV infection during infancy |  | Unadjusted model (n=291) |  | Adjusted model (n=275)^‡^ |
| RSV infection during infancy with the RSV-A G_dup–_ genotype |  | Reference |  | Reference |
| RSV infection during infancy with the RSV-B G_dup+_ genotype |  | 0.19 (-0.43, 0.81) |  | 0.26 (-0.40, 0.92) |
| RSV infection during infancy with the RSV-A G_dup+_ genotype |  | 0.35 (-0.27, 0.97) |  | 0.42 (-0.24, 1.09) |
| Overall p-value |  | 0.54 |  | 0.45 |
| *Definition of abbreviations:* G_dup_ = G gene sequence duplication, RSV = Respiratory syncytial virus.  ^*^Table estimates were obtained from unadjusted and adjusted linear regression models and are shown as β coefficient (95% confidence interval). The overall p-values for the categorical exposure term and the total number of children included in each model (n) are also shown. For all models, the reference group included children with RSV infection during infancy with the RSV-A G_dup–_ genotype. The respiratory severity score is an ordinal scale from 0-12 with higher values indicating more severe disease.  ^†^Statistical analyses were conducted in children with complete data.  ^‡^The adjusted model included the child’s age at RSV infection during infancy, sex, maternal asthma, race and ethnicity, ever breastfeeding, and daycare attendance during infancy as covariates. | | | | |
