# Supplementary material for: Infant Infection With Respiratory Syncytial Virus Genotypes and Subsequent Childhood Asthma Risk
Source: J Infect Dis. 2026 Mar 3;234(1):e34–9. doi: 10.1093/infdis/jiag104 (PMC13431657; doi:10.1093/infdis/jiag104)
Supplement: jiag104_Supplementary_Data [file jiag104_supplementary_data.zip › crs_rsv_gdup_supplementary_table_4_R1.docx]

| **Supplementary Table 4.** The association of genotype of the RSV infection during infancy with 5-year current asthma phenotype.^*†^ | | | | |
| --- | --- | --- | --- | --- |
| Genotype of the RSV infection during infancy |  | Unadjusted model (n=774) |  | Adjusted model (n=771)^‡^ |
| **Non-allergic asthma** | | | | |
| No RSV infection during infancy |  | Reference |  | Reference |
| RSV infection during infancy with the RSV-A G_dup–_ genotype |  | 0.50 (0.07, 3.78) |  | 0.46 (0.06, 3.58) |
| RSV infection during infancy with the RSV-B G_dup+_ genotype |  | 2.18 (1.00, 4.78) |  | 2.14 (0.96, 4.78) |
| RSV infection during infancy with the RSV-A G_dup+_ genotype |  | 3.56 (1.80, 7.04) |  | 3.62 (1.79, 7.32) |
| **Allergic asthma** | | | | |
| No RSV infection during infancy |  | Reference |  | Reference |
| RSV infection during infancy with the RSV-A G_dup–_ genotype |  | 1.35 (0.46, 3.98) |  | 1.02 (0.33, 3.13) |
| RSV infection during infancy with the RSV-B G_dup+_ genotype |  | 1.14 (0.50, 2.64) |  | 1.16 (0.49, 2.75) |
| RSV infection during infancy with the RSV-A G_dup+_ genotype |  | 0.86 (0.33, 2.23) |  | 0.86 (0.32, 2.29) |
| Overall p-value |  | 0.02 |  | 0.02 |
| *Definition of abbreviations:* CI = Confidence interval, G_dup_ = G gene sequence duplication, OR = Odds ratio, RSV = Respiratory syncytial virus.  ^*^Table estimates were obtained from unadjusted and adjusted multinomial logistic regression models and are shown as OR (95%CI). The overall p-values for the categorical exposure term and the total number of children included in each model (n) are also shown. The overall p-values for the categorical exposure term were calculated using the likelihood ratio test. For all models, the reference group included children without RSV infection during infancy.  ^†^Statistical analyses were conducted in children with complete data.  ^‡^The adjusted model included the child’s sex, race and ethnicity, maternal asthma, ever breastfeeding, and daycare attendance during infancy as covariates. | | | | |
