# Supplementary figures and images for: Infant Infection With Respiratory Syncytial Virus Genotypes and Subsequent Childhood Asthma Risk
Source: J Infect Dis. 2026 Mar 3;234(1):e34–9. doi: 10.1093/infdis/jiag104 (PMC13431657; doi:10.1093/infdis/jiag104)

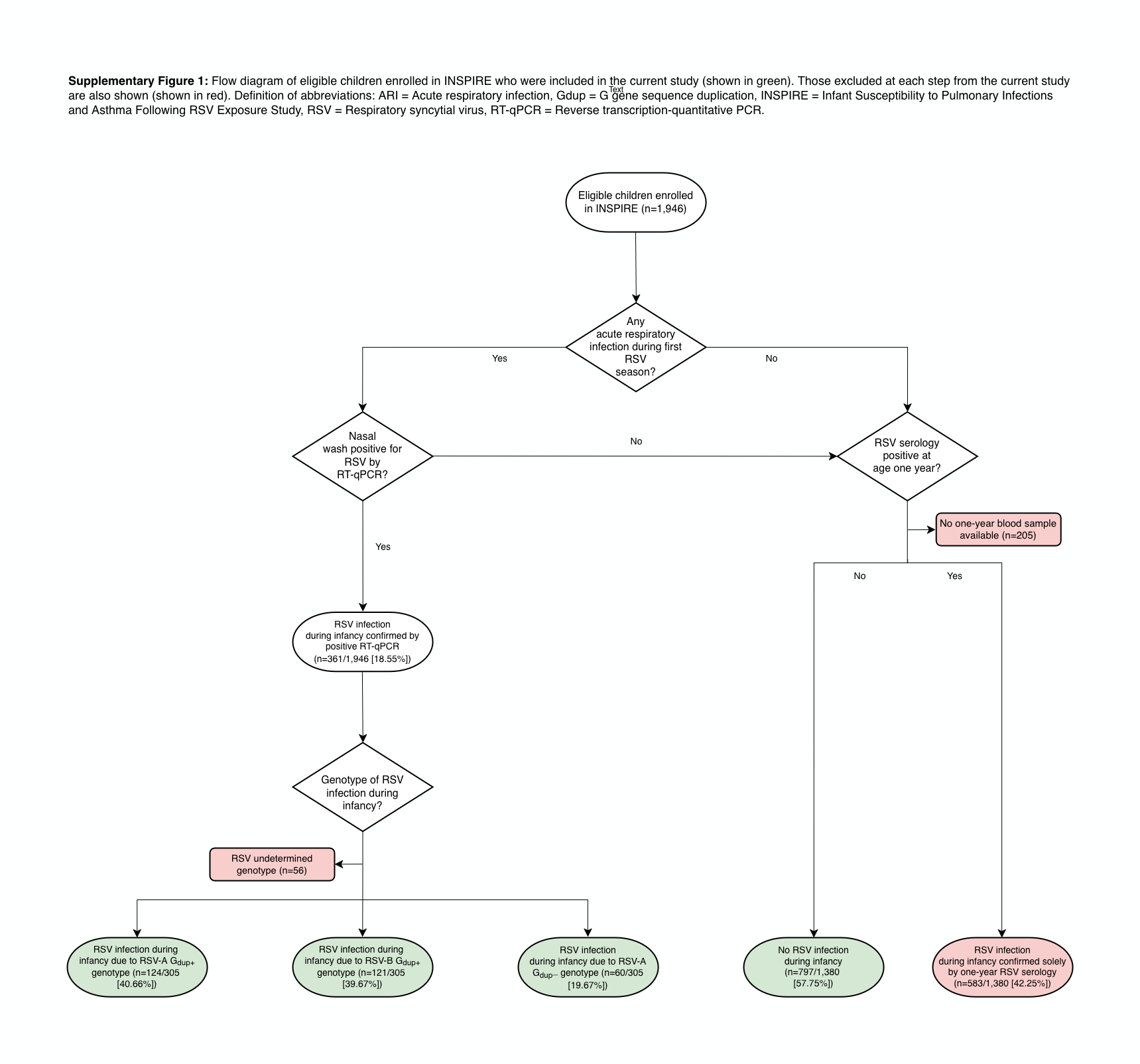

Supplement: jiag104_Supplementary_Data [file jiag104_supplementary_data.zip › crs_rsv_gdup_supplementary_figure_1_R1.tiff]

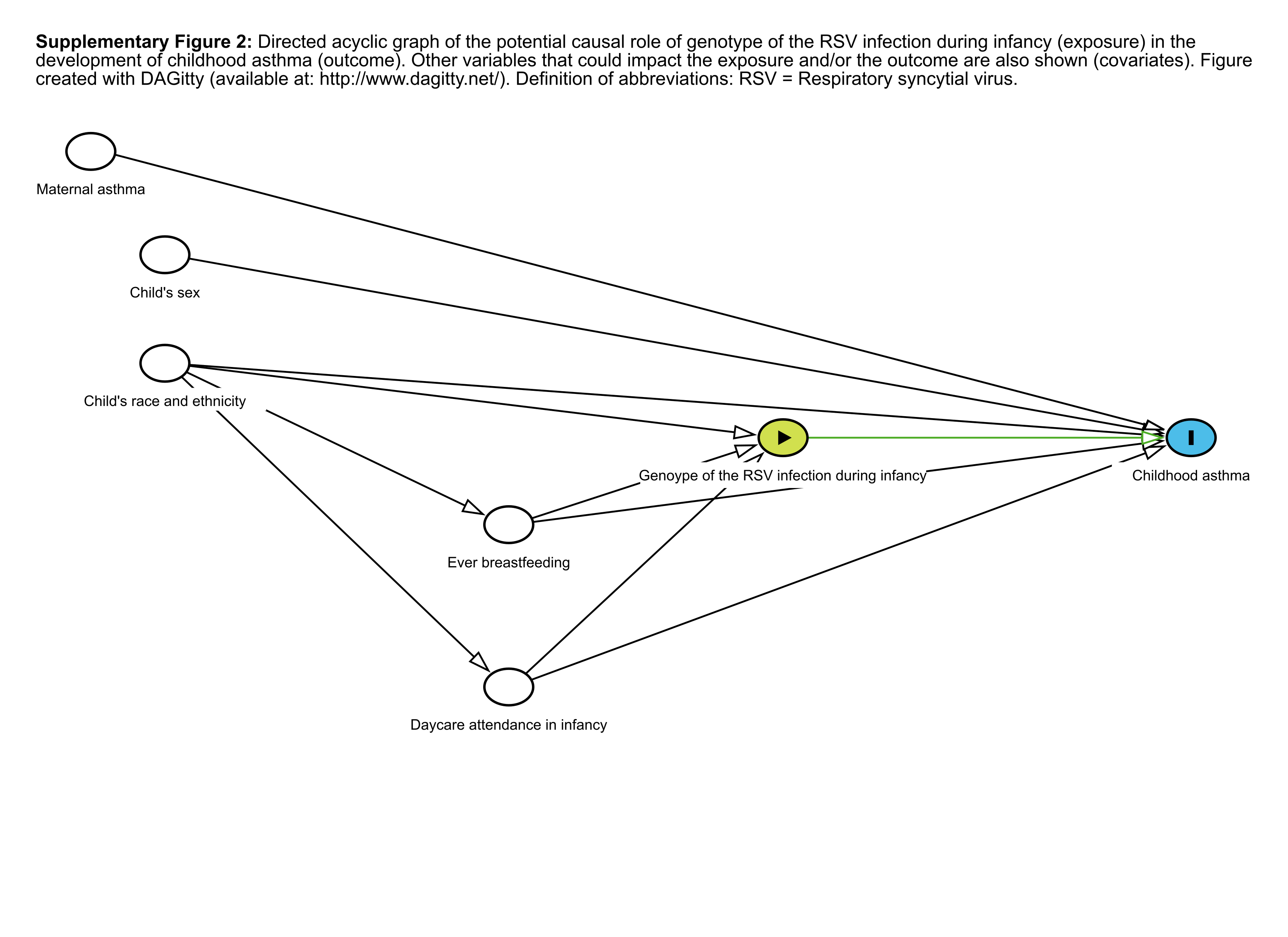

Supplement: jiag104_Supplementary_Data [file jiag104_supplementary_data.zip › crs_rsv_gdup_supplementary_figure_2_R1.tiff]

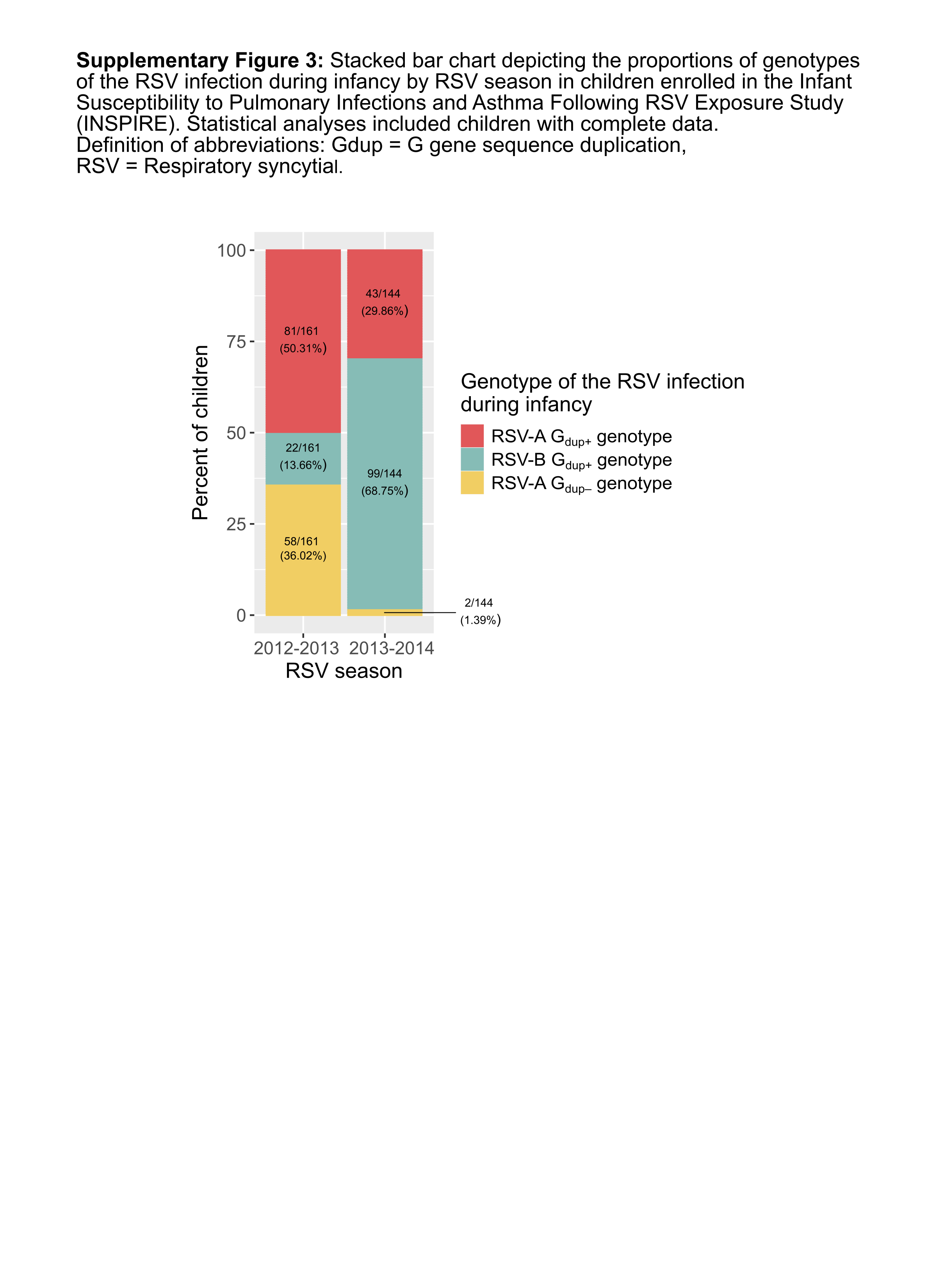

Supplement: jiag104_Supplementary_Data [file jiag104_supplementary_data.zip › crs_rsv_gdup_supplementary_figure_3_R1.tiff]
